# Glucose Uptake and Oxidative Stress in Caco-2 Cells: Health Benefits from *Posidonia oceanica* (L.) Delile

**DOI:** 10.3390/md20070457

**Published:** 2022-07-14

**Authors:** Camilla Morresi, Marzia Vasarri, Luisa Bellachioma, Gianna Ferretti, Donatella Degl′Innocenti, Tiziana Bacchetti

**Affiliations:** 1 Department of Clinical Experimental Science and Odontostomatology-Biochemistry, Università Politecnica delle Marche, 60100 Ancona, Italy; m.morres@libero.it (C.M.); g.ferretti@univpm.it (G.F.); 2 Department of Experimental and Clinical Biomedical Sciences, University of Florence, Viale Morgagni 50, 50134 Florence, Italy; marzia.vasarri@unifi.it; 3 Department of Life and Environmental Sciences-Biochemistry, Università Politecnica delle Marche, 60100 Ancona, Italy; luisabellachioma@gmail.com (L.B.); t.bacchetti@univpm.it (T.B.); 4 Interuniversity Center of Marine Biology and Applied Ecology “G. Bacci” (CIBM), Viale N. Sauro 4, 57128 Livorno, Italy

**Keywords:** *Posidonia oceanica*, AGEs, marine antioxidants, oxidative stress, intestinal glucose uptake, intestinal barrier integrity

## Abstract

*Posidonia oceanica* (L.) Delile is an endemic Mediterranean marine plant of extreme ecological importance. Previous in vitro and in vivo studies have demonstrated the potential antidiabetic properties of *P. oceanica* leaf extract. Intestinal glucose transporters play a key role in glucose homeostasis and represent novel targets for the management of diabetes. In this study, the ability of a hydroalcoholic *P. oceanica* leaf extract (POE) to modulate intestinal glucose transporters was investigated using Caco-2 cells as a model of an intestinal barrier. The incubation of cells with POE significantly decreased glucose uptake by decreasing the GLUT2 glucose transporter levels. Moreover, POE had a positive effect on the barrier integrity by increasing the Zonulin-1 levels. A protective effect exerted by POE against oxidative stress induced by chronic exposure to high glucose concentrations or tert-butyl hydroperoxide was also demonstrated. This study highlights for the first time the effect of POE on glucose transport, intestinal barrier integrity, and its protective antioxidant effect in Caco-2 cells. These findings suggest that the *P. oceanica* phytocomplex may have a positive impact by preventing the intestinal cell dysfunction involved in the development of inflammation-related disease associated with oxidative stress.

## 1. Introduction

*Posidonia oceanica* (L.) Delile is a seagrass of extreme ecological importance for the whole marine ecosystem, and it is the only species of the Posidoniaceae family endemic in the Mediterranean Sea [1,2]. Although *P. oceanica* leaves have been used in ancient times in traditional medical practices as a natural remedy for various health disorders, only more recent scientific studies have described its potential benefits for human health, including an anti-diabetic property [3]. For instance, Gokce et al. (2008) demonstrated that *P*. *oceanica* leaf extract has hypoglycemic properties in alloxan-induced diabetic rats [4]. Oral administration of its extract for 15 days dose-dependently decreased blood glucose in diabetic rats [4]. In addition, the hydroalcoholic extract of *P. oceanica* leaves (POE) was able to inhibit the in vitro glucose-induced glycation of human serum albumin [5]. POE has also demonstrated antioxidant and anti-inflammatory properties in in vitro cellular models and in vivo animal models [6,7].

**Figure 1:**
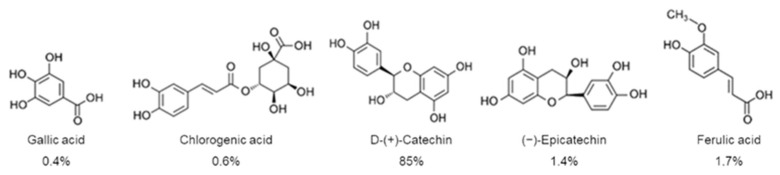
Phenolic compounds with relative percentages identified in *P. oceanica* leaf extract (POE) by UPLC analysis [8].

Overall, the POE biological effects have been related to the synergistic action of its individual constituents. A first UPLC characterization analysis showed that POE consists of 88% phenolic compounds, mostly represented by D-(+)-catechin, and less by gallic acid, ferulic acid, (−)-epicatechin, and chlorogenic acid [8] (Figure 1).

Among the biological properties of dietary polyphenols, growing attention has been devoted to their ability to modulate post-prandial increases in glucose levels and to modulate intestinal integrity and oxidative damage [9,10,11,12,13,14]. In fact, high post-prandial plasma glucose concentrations are associated with an increased risk of developing type 2 diabetes (T2D) and metabolic syndrome [15]. Intestinal glucose transporters such as sodium glucose co-transporter-1 (SGLT1) and glucose transporter 2 (GLUT2) play a role in glucose homeostasis and represent targets for the management of diabetes [16].

A Caco-2 cell monolayer is one of the most widely used in vitro model of the human intestinal barrier to study absorption [17,18]. This cellular model was used in this study to evaluate the effect of POE on the intestinal glucose uptake and its ability to modulate the levels of glucose transporters (SGLT1 and GLUT2). Furthermore, the intestinal barrier integrity is essential for the metabolic homeostasis. A dysfunction of the intestinal barrier is linked to inflammatory and dysmetabolic conditions, including diabetes [19]. 

In this study, the effect of POE on intestinal barrier integrity was verified by assessing transepithelial electrical resistance (TEER) across a monolayer of intestinal Caco-2 cells. The effect of POE on the level of Zonula occludens-1 (ZO-1), a protein involved in the regulation of intestinal barrier integrity, was also evaluated.

In addition, the potential protective effect of POE against oxidative stress has been studied in Caco-2 cells using tert-butyl hydroperoxide (TBHP) or chronic exposition to high glucose (HG) levels.

## 2. Results

### 2.1. Biochemical Characterization of P. oceanica Leaf Extract (POE)

POE was found to contain 3.4 ± 0.2 mg/mL of total polyphenols (TP) equivalent to gallic acid (mg GAE/mL). In addition, POE exhibited antioxidant and radical-scavenging activities of 0.9 ± 0.2 mg/mL and 8.9 ± 0.3 mg/mL ascorbic acid equivalents (mg AAE/mL), as evaluated by FRAP and DPPH assays, respectively (Table 1). The data obtained are consistent with those obtained in previous work [6,8], supporting the efficiency and reproducibility of the extraction method.

### 2.2. Effect of POE on Cell Viability 

No significant modifications of cell viability were observed in Caco-2 cells treated till to 24 h with increasing levels of POE (corresponding to polyphenol concentration ranging from 5 μg GAE/mL to 40 μg GAE/mL) (Supplementary Figure S1). These results confirm that POE did not exert a cytotoxic effect in our experimental conditions. Based on these results, all the following experiments were conducted using 15 μg GAE/mL POE.

### 2.3. Effect of POE on Glucose Transport under Sodium-Dependent or Sodium-Free Conditions

Caco-2 differentiated cells were used as a model of the intestinal barrier. The effect of POE on glucose uptake was investigated both in the presence or in the absence of sodium. In the presence of sodium, glucose transporters SGLT1 and GLUT2 are both active. In sodium-free conditions only GLUT2 is active.

**Figure 2:**
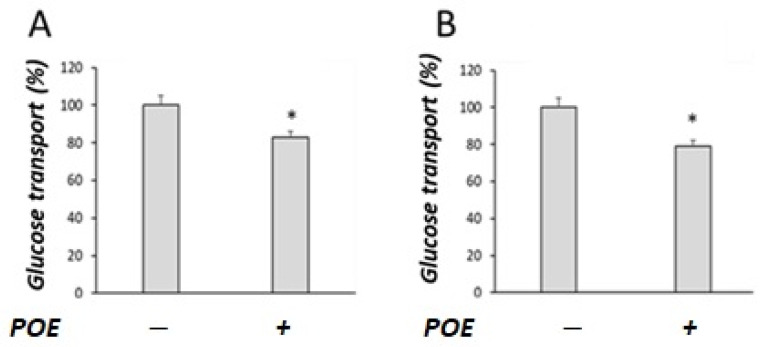
Effect of POE on glucose transport in Caco-2 differentiated cells. Glucose transport was evaluated under (**A**) sodium-dependent or (**B**) sodium-free conditions in differentiated Caco-2 cells treated with POE (15 µg GAE/mL) for 24 h. Values are presented as the mean ± SD of three determinations carried out in triplicate. Data are reported in terms of percentage with respect to control cells. *: *p* < 0.05.

As shown in Figure 2, POE treatment significantly (*p* < 0.05) decreased glucose transport, both in the presence (Figure 2A) and absence of sodium (Figure 2B).

**Figure 3:**
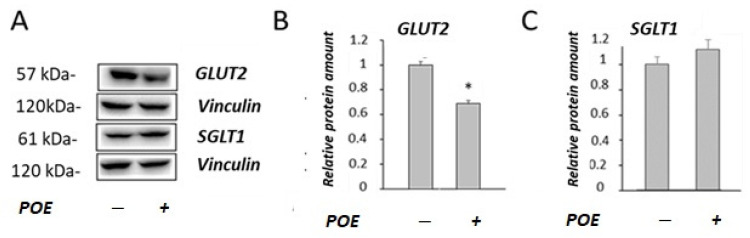
Effect of POE on the levels of glucose transporters. (**A**) Representative Western blot images of (**B**) GLUT2 and (**C**) SGLT1 glucose transporters in differentiated Caco-2 cells incubated in the absence or presence of POE (15 µg GAE/mL) for 24 h. Densitometric data are normalized to the Vinculin expression levels. Data are presented as the mean ± SD of three determinations. *: *p* < 0.05.

To understand the decrease in glucose transport observed in POE-treated cells, the effect of POE on the expression of glucose transporters (SGLT1 and GLUT2) in Caco-2 differentiated cells was investigated by Western blot analysis (Figure 3A). POE treatment caused a significant (*p* < 0.05) decrease in the GLUT2 levels (68% ± 2.1%) with respect to control cells, as shown in Figure 3B. No modifications of the levels of transporter SGLT1 were observed in POE-treated cells compared with control cells (Figure 3C).

### 2.4. Effect of POE on Caco-2 Monolayer as a Model of Intestinal Barrier 

The evaluation of transepithelial electrical resistance (TEER) across the monolayer of Caco-2 differentiated cells was used to assess the effect of POE on the integrity of the intestinal barrier. 

**Figure 4:**
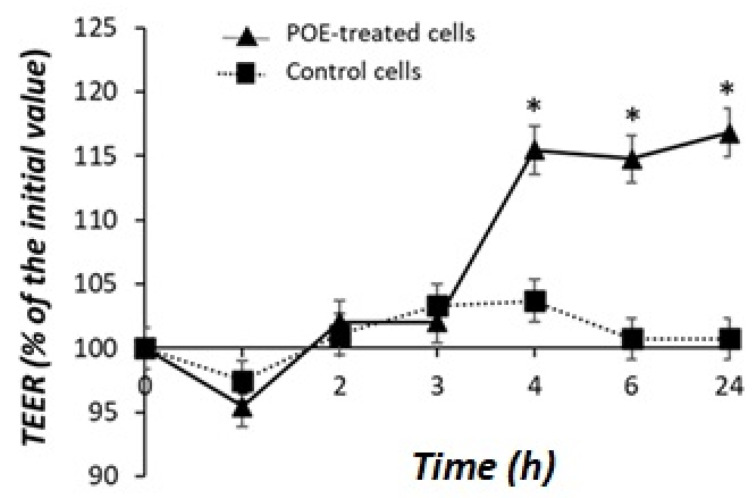
Effect of POE on Caco-2 monolayer cells. Transepithelial electrical resistance (TEER) in differentiated Caco-2 control cells or treated with POE (15 µg GAE/mL) for 24 h. Values are presented as the mean ± SD of three determinations carried out in triplicate. TEER values are reported in terms of percentage with respect the initial value. * Control vs. POE-treated cells. (*: *p* < 0.05).

As shown in Figure 4, POE treatment caused an increase in TEER across the Caco-2 cell monolayer. The effect was significant (*p* < 0.05) after 4 h and reached 118% of the initial value after 24 h of incubation compared with control cells.

**Figure 5:**
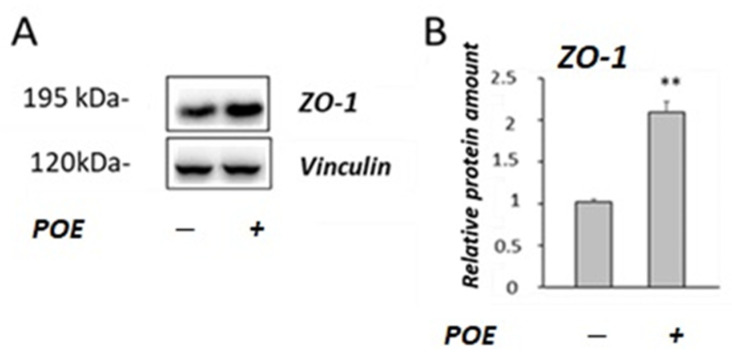
Effect of POE on the ZO-1 levels. (**A**) Representative Western blot images of ZO-1 in differentiated Caco-2 incubated in the absence or treated with POE (15 µg GAE/mL) for 24 h. (**B**) Densitometric data are normalized to Vinculin. Data are presented as the mean ± SD of five determinations. **: *p* < 0.01.

Zonulin-1 (ZO-1) is a protein bound to the cytoskeleton and it has a pivotal role in tight junction (TJ) integrity. Therefore, to investigate the molecular mechanisms involved in the POE-induced increase in TEER, the levels of ZO-1 were determined by Western blot analysis (Figure 5A).

As shown in Figure 5B, a two-fold increase in the ZO-1 levels was observed in POE-treated Caco-2 cells compared with the untreated control cells (*p* < 0.01).

### 2.5. Effect of POE on High-Glucose-Induced Oxidative Stress

The chronic exposure to high glucose (HG) induces oxidative stress in Caco-2 cells, as previously described [20]. 

**Figure 6:**
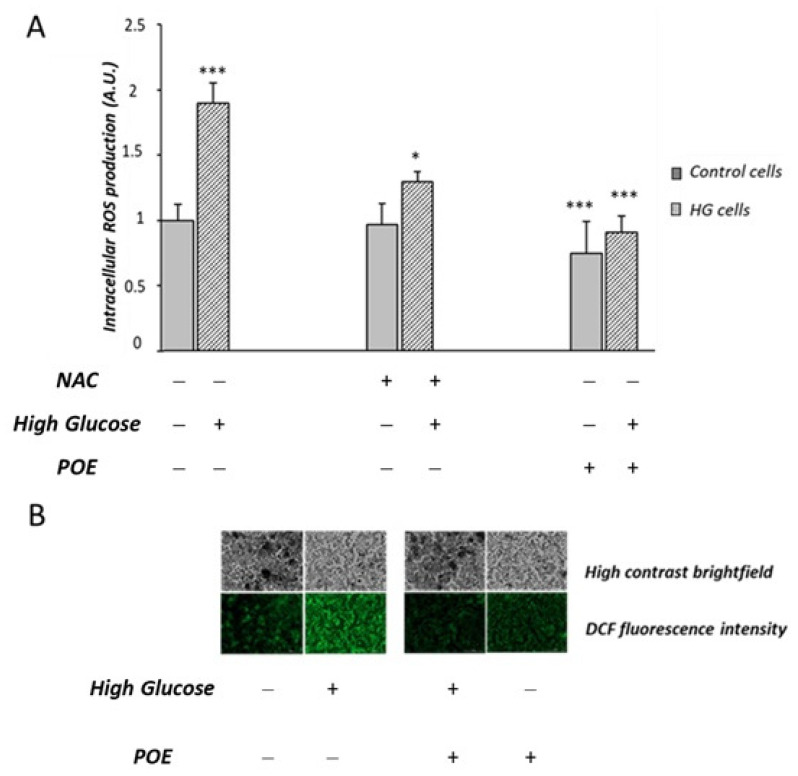
Effect of POE and *N*-acetylcysteine (NAC) on HG-induced intracellular ROS formation. (**A**) Intracellular ROS production was evaluated in Caco-2 cells incubated in the absence (control cells) or in the presence of high-glucose conditions (HG cells) for 1 week and co-incubated with POE (15 µg GAE/mL), and *N*-acetylcysteine (NAC) (50 µM) for the last 24 h. Values are represented as the mean ± SD of five determinations carried out in triplicate. *** Control vs. HG; * HG vs. HG + NAC; *** HG vs. POE; *** HG vs. HG + POE. (ANOVA: *: *p* < 0.05; ***: *p* < 0.001). (**B**) Fluorescent cells observed under a fluorescent microscope in the presence of POE (15 µg GAE/mL (Lionheart™ FX)).

In this study, the potential protective role of POE (15 µg GAE/mL) on chronic HG-induced oxidative stress was investigated in Caco-2 cells. Cells not exposed to HG were used as control cells. A significant increase (1.95 ± 0.7 A.U.) in intracellular ROS production was confirmed in HG cells compared to control cells (1.02 ± 0.18 A.U.), in accordance with our previous study [20]. HG cells treated with POE showed a significant decrease in intracellular ROS production with respect to HG cells (Figure 6A). Figure 6B shows representative images of the fluorescence intensity indicative of ROS formation in POE-treated cells under HG conditions. The dose-dependent effect (0–40 µg GAE/mL) of POE on HG-induced intracellular ROS formation is reported in Supplementary Figure S2. 

HG cells treated with *N*-acetylcysteine (NAC) were used as the positive antioxidant control. The POE-induced mitigation of intracellular ROS production in HG cells was comparable to that induced by NAC (50 µM) (Figure 6A). 

This finding suggests that POE exerts an antioxidant activity against HG-induced intracellular ROS production in Caco-2 cells. 

The HG-induced intracellular ROS levels can cause an increase in advanced glycation end products (AGEs), due to the formation of highly reactive intermediates of the Maillard reaction, such as glycolaldehyde and glyoxal. These molecules are involved in cross-linking of proteins and are precursors of AGEs [21]. 

**Figure 7:**
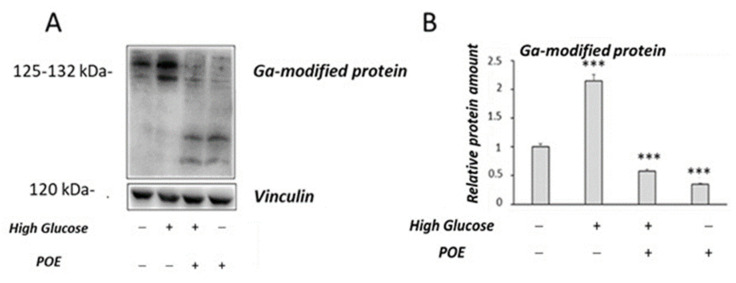
Effect of POE on GA-modified proteins levels in Caco-2 cells. (**A**) Representative Western blot images of GA-modified proteins. (**B**) Densitometric analysis of GA-modified proteins in Caco-2 control cells or HG cells in the absence or presence of POE (15 µg GAE/mL) for 24 h. Densitometric data are normalized to Vinculin. Results are presented as the mean ± SD of five determinations. *** Control vs. HG; *** Ctrl vs. POE; *** HG vs. POE; *** HG vs. HG + POE (ANOVA: ***: *p* < 0.001).

In this study, the glycolaldehyde (GA)-modified protein levels were also evaluated by Western blot analysis (Figure 7A) to investigate the effect of POE on AGEs formation in HG-treated cells. Higher levels of GA-modified proteins were observed in HG cells compared to control cells (Figure 7B), as previously described [20]. In the presence of POE, a significant (*p* < 0.05) decrease in GA-modified protein levels was observed in HG cells compared with untreated HG cells (Figure 7B). A slight decrease in the GA-modified protein levels was also observed in Caco-2 cells treated with POE compared with the untreated control cells (Figure 7B).

These results could explain the effect of POE on HG-induced cell toxicity. In fact, a significant decrease (about 40%) in cell viability was observed in HG cells compared with control cells. POE-treated cells exposed to chronic HG conditions had comparable viability to control cells (Supplementary Figure S3)

### 2.6. Effect of POE on Tert-Butyl Hydroperoxide-Induced Oxidative Stress

To further verify the potential antioxidant role of POE against intracellular ROS formation, the effect of POE was also investigated in Caco-2 cells acutely treated with the pro-oxidant agent tert-butyl hydroperoxide (TBHP). 

**Figure 8:**
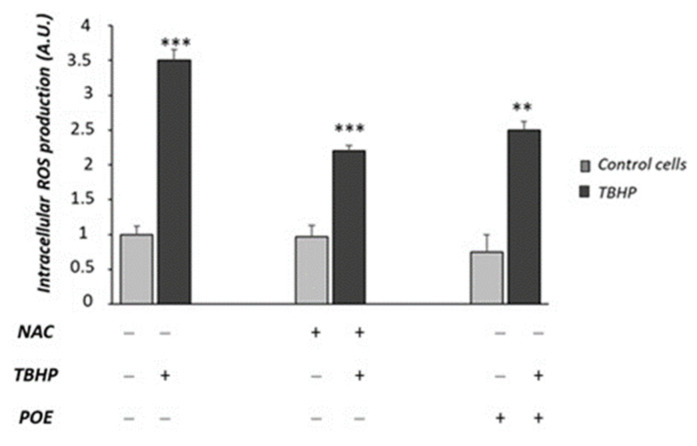
Effect of POE on TBHP-induced ROS formation in Caco-2 cells. Intracellular ROS production was evaluated in Caco-2 cells pre-incubated for 24 h with POE (15 µg GAE/mL) or NAC (50µM) and treated with TBHP (50 µM) for 90 min. Values are represented as the mean ± SD of five determinations carried out in triplicate. *** Control vs. TBHP; *** NAC + THBP vs. TBHP; ** POE + THBP vs. TBHP (ANOVA: **: *p* < 0.01; ***: *p* < 0.001).

As shown in Figure 8, TBHP induced a more than three-fold increase in intracellular ROS production with respect to control cells (*p* < 0.001). NAC was able to reduce intracellular ROS production in TBHP-treated cells (*p* < 0.001). POE showed a comparable effect to NAC in TBHP-treated cells (Figure 8).

## 3. Discussion

The Caco-2 cell line is extensively used as a model of the intestinal epithelial barrier. In particular, monolayers of differentiated Caco-2 cells are used for the investigation of the function and integrity of the intestinal barrier. Therefore, this cell model is often used to study the modulatory role that natural compounds (including polyphenols) may have on intestinal function [17,18]. Here, for the first time, POE, a polyphenol-rich phytocomplex, has been shown to inhibit glucose uptake, improve intestinal barrier integrity, and protect cells from oxidative stress in Caco-2 cells. 

The inhibition of glucose uptake induced by POE was observed to the same extent in both the presence and absence of sodium. Moreover, a significant decrease in the GLUT2 levels in POE-treated cells was observed in our experimental conditions. On the contrary, no effect of POE on the SGLT1 protein levels was observed. These results suggest that the effect of POE on glucose could be mainly mediated by a modulation of the expression of glucose transporter GLUT2, in agreement with other studies that reported a reduction in the expression of intestinal glucose transporters by polyphenols [11]. In rat enterocytes, the apical membrane levels of both transporters can alter rapidly in response to cell signaling events [21,22]. GLUT2 has been detected at both the apical membrane and basolateral membrane of Caco-2 cells [23]. Further studies are required to address the effect of POE on cell signaling events and the cellular distribution of GLUT2 and SGLT1.

Elevated levels of intestinal glucose transporters have been reported in diabetic and obese animal models and this contributed directly to their hyperglycemic status [24,25]. Therefore, compounds that regulate glucose transporter expression may be useful as potential anti-hyperglycemic agents. Till today, this effect has been observed only in animal models. Gokce et al. (2008) have reported that the oral administration of a *P. oceanica* extract for 15 days (50, 150, and 250 mg/kg b.wt.) resulted in a dose-dependent decrease in blood glucose in alloxan-induced diabetic rats [4]. Further studies are needed to investigate the effects of POE intake on post-prandial glycaemia in normal and hyperglycemic subjects.

A dysfunctional intestinal barrier is associated also with dysmetabolic diseases, including diabetes and obesity [19,26,27]. Therefore, in this study, transepithelial electrical resistance (TEER) [28] and the levels of TJ proteins, such as Zonula occludens (ZO-1), have been evaluated to investigate the potential role of POE on the integrity of cell monolayer using differentiated Caco-2 cell, as a model of an intestinal barrier. A significant increase in TEER across the cellular monolayer in POE-treated cells was observed with respect to untreated control cells. Furthermore, POE demonstrated a positive effect on intestinal cells by increasing the levels of ZO-1. The protein ZO-1 is involved in the regulation of intestinal barrier integrity and plays a crucial role as a key molecule in cell-to-cell contact and in maintaining the structure of TJ and the epithelial barrier function [26]. TJ barrier integrity is essential for human health and metabolic homeostasis [26]. The effects of POE on ZO-1 are in agreement with previous studies, which demonstrated that polyphenols (such as catechins and phenolic acids) modulate intestinal barrier function and increase the expression of several TJ proteins, including ZO-1, in in vitro models [12,13]. Some in vivo studies have demonstrated that, in older subjects, a polyphenol-rich dietary pattern improves intestinal permeability, evaluated as the serum Zonulin levels [29]. However, further in vivo studies will be required to verify the protective effect of POE on the intestinal barrier integrity.

Intestinal cells are exposed to dietary pro-oxidants, AGEs, lipid peroxidation products, and are susceptible to oxidative damage [30,31,32]. In addition, previous studies have also shown that high-sugar diets cause increased oxidative stress and inflammation [31]. 

ROS-induced oxidative stress is widely considered as a possible upstream mechanism of high-glucose-induced cell damage [20]. These cellular alterations may cause a dysfunction of intestinal barrier and lead to the onset of the intestinal bowel diseases [30,31]. Previous studies have reported that POE has an antioxidant role against intracellular ROS formation in macrophages activated by LPS [6]. In our experimental conditions, POE shows a protective role against ROS-induced oxidative stress under chronic high-glucose conditions in Caco-2 cells. The antioxidant role of POE was also confirmed by an inhibitory action against the ROS formation induced, in the same cell model, by treatment with TBHP, a molecule commonly used to study cellular alterations resulting from oxidative stress [33].

Chronic exposure to high glucose evokes oxidative stress; the resulting high levels of intracellular ROS can promote AGEs formation. Indeed, previous studies have shown that highly reactive intermediates of the Maillard reaction, such as glycolaldehyde (GA) and glyoxal, are involved in cross-linking of proteins and are precursors of AGEs [34]. In this study, Caco-2 cells treated with POE showed a significant decrease in the GA protein levels in chronic high-glucose conditions. Since GA proteins are useful markers of oxidative stress [20], our findings further support the protective role of POE against oxidative stress induced by high glucose. Overall, these results are in agreement with our previous studies that described an in vitro role of POE against glucose-induced glycation of human serum albumin [5]. Moreover, other studies have demonstrated the ability of polyphenols to exert a protective effect against oxidative stress and formation of AGEs in Caco-2 cells [14]. In addition, previous studies have reported that some phenolic compounds exert a protective effect against oxidative stress, either by reducing ROS production during the glycation process or by trapping of dicarbonyl species [35].

In conclusion, the data reported in this study demonstrate that POE reduces glucose transport by lowering the GLUT2 levels and promotes intestinal barrier integrity by positively modulating the ZO-1 levels. Furthermore, POE has a protective antioxidant effect against high-glucose-induced damage, in terms of lower production of intracellular ROS and AGE-modified proteins.

Inhibition of glucose uptake in the small intestine may prevent post prandial hyperglycemia, which is one of the risk factors for diabetes and metabolic syndrome. Our findings suggest that POE may have a positive impact by preventing the intestinal cell dysfunction involved in the development of inflammation-related intestinal diseases associated with oxidative stress. 

The in vitro effect of POE was observed at a concentration of polyphenols of 15 µg GAE/mL (88 µmol/L). It has been reported that the bioavailability of polyphenols is related to the structural properties of molecules. The total levels of polyphenols are present in plasma at <1 µmol/L concentrations, but they are present in the stomach and intestinal lumen at much higher concentrations after consumption of vegetables rich in polyphenols. Saura-Calixto et al. (2007) demonstrated that polyphenols could act as antioxidants in the intestine because they are present at millimolar concentrations after consumption of fruits and vegetables [36].

## 4. Materials and Methods

### 4.1. Reagents

Reagents for cell culture were obtained by Euroclone (Euroclone, Italy). All chemical reagents were purchased by Sigma Aldrich (Sigma, St. Louis, MO, USA). 2′,7′-Dichlorodihydrofluorescein diacetate (H_2_DCFDA) was supplied by Invitrogen (Invitrogen, Carlsbad, CA, USA).

### 4.2. Preparation and Characterization of Posidonia oceanica (L.) Delile Extract

The hydroalcoholic extract of *P. oceanica* (POE) was obtained according to a previously described method [8]. Briefly, 1.84 mg of the dry extract from *P. oceanica* leaves were resuspended in 500 μL of 70% ethanol; POE was diluted and used in different experimental conditions. 

### 4.3. Evaluation of Total Polyphenols in POE

Total polyphenol (TP) content in POE was evaluated using Folin–Ciocalteu’s method, as previously described [8]. Briefly, for the determination of TP in POE, 100 μL of Folin–Ciocalteu (Folin–Ciocalteu phenol reagent diluted 1:10 in H_2_O) was added to scalar volumes of POE. After a 5 min incubation at room temperature, 80 μL of 7.5% sodium carbonate solution was added for another 2 h. Absorbance at 595 nm was recorded with a microplate reader. Gallic acid (0.5 mg/mL) was used as a reference to determine the TP values. TP is expressed as mg of gallic acid equivalents (GAE) per mL. 

### 4.4. Evaluation of Antioxidant and Radical Scavenging Activities of POE 

The antioxidant and radical scavenging activities of POE were studied using ferric reducing/antioxidant potency (FRAP) and the 2,2-diphenyl-1-picrylhydrazyl (DPPH) radical [8]. Briefly, for determination of the antioxidant activity of POE by FRAP, 200 μL of Ferrozine™ reagent (10 mM Ferrozine™ in 40 mM HCl: 20 mM FeCl_3_: 0.3 M acetate buffer pH 3.6 1:1:10 ratio) was added to scalar volumes of POE. After 4 min incubation at 37 °C, absorbance was measured at 595 nm at room temperature using a microplate reader. 

For determination of the radical scavenging activities of POE, 100 μL of 95% methanol was added to scalar volumes of POE and mixed with 100 μL of freshly prepared DPPH solution (0.15 mg/mL in methanol). After a 30 min incubation in the dark at room temperature, absorbance was read at 490 nm with a microplate reader. Ascorbic acid (0.1 mg/mL) was used as a reference to determine the values of both the antioxidant and radical scavenging activity. These activities are expressed as mg of ascorbic acid equivalents (AAE) per mL.

### 4.5. Caco-2 Cells

Human colon epithelial cells, Caco-2 (ATCC^®^HTB-37™), were purchased from the American Type Culture Collection (Rockville, MD, USA). In our experiments, the Caco-2 cells were cultured in Dulbecco’s Minimal Essential Medium (DMEM) supplemented with 10 mM nonessential amino acids, 10% (*v*/*v*) fetal bovine serum (FBS), 100 μg/mL streptomycin, 100 U/mL penicillin, and 2 mM glutamine (growth medium). Cells were incubated at 37 °C and 5% CO_2_, and sub-cultured at 80–90% confluence every 3–4 days.

For differentiation, Caco-2 cells were seeded at a density of 1 × 10^5^ cells/well in 12-well trans-well plate (12 mm, with 0.4 µm pore polycarbonate membrane Insert, Corning) and differentiated for 21 days in DMEM growth medium. The medium was replaced every 2–3 days for both the apical (AP) and basal (BL) sides of the trans-well filters. The integrity of cell monolayer was checked by measuring the trans-epithelial electrical resistance (TEER) before and after the experiments with an Epithelial Volt/Ohm Meter (EVOM).

### 4.6. Cell Viability Assay 

Cell viability in Caco-2 cells treated in different experimental conditions was analyzed following the enzymatic reduction of 3-[4,5-dimethylthiazole-2-yl]-2,5-diphenyltetrazolium bromide (MTT) to MTT-formazan, catalyzed by mitochondrial succinate. Briefly, 100 μL of MTT solution (5 mg/mL) was added to each well. After 2 h, the incubation buffer was removed and the blue MTT–formazan product was extracted with DMSO (dimethyl sulfoxide). Supernatants were collected in a 96-well plate and the absorbance was measured at 540 nm (Microplate Rader) [37].

### 4.7. Evaluation of Glucose Transport in Caco-2 Cells

Glucose transport was evaluated in differentiated Caco-2 cells as described by Sharma et al. (2020) [12], applying some modifications. The transport was analyzed both in a sodium and non-sodium environment. Caco-2 cells were washed 2 times in HBBS (pH 7.5). Subsequently, POE (15 μg/mL) was co-incubated for 24 h in the apical compartment with 5 mM D-glucose prepared in DPBS. In the basolateral compartment was added DPBS. Similarly, sodium-free buffer was used for the sodium independent absorption experiment. Cells were incubated at 37 °C and 5% CO_2_ for the duration of the experiment. To follow transport across the cell monolayer, 50 μL was collected in the basolateral compartment at time 0 and after 24 h. Glucose transport was measured using 65.8 mM of *p*-hydroxyacetophenonebenzoylhydrazone (PAHBH) added to the collected samples (30:1). The mixture was incubated for 10 min at 90 °C and then read in a 96-well plate reader at 410 nm. Glucose (0.01–5 mM) was used to create the standard curve. Data were expressed as % of glucose transport with respect to each specific control [38].

### 4.8. Evaluation of Caco-2 Monolayer Permeability

Monolayers of Caco-2 differentiated cells were incubated for 24 h in the presence or absence of POE (15 μg GAE/mL). Resistance measurement was performed by TEER. Cells were incubated at 37 °C and 5% CO_2_ for the duration of the experiment. TEER was measured using an EVOM with a chopstick electrode (Millicell ERS-2, EMD Millipore, Billerica, MA, USA). The electrode was immersed at a 90° angle with one tip in the basolateral chamber and the other in the apical chamber. Care was taken to avoid electrode contact with the monolayer and triplicate measurements were recorded for each monolayer. An insert without cells was used as a blank and its mean resistance was subtracted from all samples. TEER measurements were registered hourly for 24 h. Results are reported as TEER % versus time [39].

### 4.9. Chronic High Glucose Caco-2 Cell Treatment 

The chronic high glucose (HG) treatment of Caco-2 cells was carried out as previously described [20]. Briefly, cells were grown in 50 mM glucose for 1 week (high glucose, HG cells). Cells grown in growth medium without addition of glucose were used as control cells (Control cells). Medium was replaced two times a week. POE (15 μg GAE/mL) or *N*-acetyl-cysteine (NAC; 50 µM) was added to the media and co-incubated (in both HG and Control conditions) for the last 24 h.

### 4.10. Tert-Butyl Hydroperoxide (TBPH) Caco-2 Cell Treatment 

Cells were pre-treated in the absence or in the presence of POE (15 µg GAE/mL) or NAC (50 µM) for 24 h. At the end of incubation cells were washed with cold PBS and incubated with TBHP (50 µM) for 90 min [40].

### 4.11. Intracellular ROS Detection

Intracellular ROS formation was evaluated in Caco-2 cells treated in the different experimental conditions using a 2′,7′-dichlorodihydrofluorescein diacetate (H_2_DCFDA) fluorescent probe [20]. Cells were incubated for 45 min with pre-warmed PBS containing the fluorescent probe (25 µM). After incubation in the dark at 37 °C, cells were washed twice in PBS. Fluorescence of labeled cells was measured in a fluorescence plate reader at λ_ex_/λ_em_ (485/535 nm) (Multi-Mode Microplate Reader Synergy^TM^ HT, BioTek Instruments, Inc.). Data obtained in cells treated in different experimental condition were normalized to the results of the cell number. Data are expressed as arbitrary units of fluorescence (A.U.). An automated microscope (Lion hearth FX Automated live cell Imager, Biotek, BioTek Instruments, Inc., Santa Clara, CA, USA) was used for cellular imaging.

### 4.12. Western Blot Analysis

Total cell lysates (50 μg proteins) from the different experimental conditions were subjected to 12.5% sodium dodecyl sulfate polyacrylamide gel electrophoresis (SDS-PAGE) and transferred onto polyvinylidene fluoride (PVDF) membranes. 

After regular blocking and washing, the membranes were incubated with specific primary antibodies over night at 4 °C.

For the determination of glucose receptors and tight junction levels: rabbit polyclonal anti-GLUT2 antibody (JJ20-21 Invitrogen, USA, diluted 1:500), rabbit polyclonal anti-SGLT1 antibody (Pa5-84085 Invitrogen, USA, diluted 1:200), and rabbit polyclonal anti ZO-1 antibody (GTX108587 GENETEX, Irvine, California, USA, diluted 1:200).

For the determination of glycolaldehyde-modified proteins (GA-modified proteins) levels: goat polyclonal anti-AGE antibody (Ab9890 Merck KGaA, Darmstadt, Germany, diluted 1:2000). Vinculin was used as the loading control (sc-25336 Santa Cruz Biotechnology, Dallas, TX, USA, diluted 1:200). 

Donkey anti-goat (AP186P Sigma-Aldrich, Merck KGaA, Darmstadt, Germany, diluted 1:100,000), goat anti-mouse (sc-2005 Santa Cruz Biotechnology, Dallas, TX, USA, diluted 1:100,000), and goat anti-rabbit (12-348Sigma-Aldrich, Darmstadt, Germany, diluted 1:150,000) secondary antibodies HRP (Horseradish Peroxidase) were used in accordance with the manufacturer’s instructions. 

Protein bands were developed by the enhanced SuperSignal West Femto Maximum Sensitivity Substrate (Thermo Fisher Scientific, Waltham, MA, USA). The chemiluminescent signal was acquired using ChemiDoc XRS+ System (Bio-Rad Laboratories, Hercules, CA, USA) and analyzed by using Image J software (Version 1.50i, National Institute of Health, Bethesda, MD, USA)**.**

### 4.13. Statistical Analysis

The experiments were performed on a minimum of three independent determinations, carried out in triplicate, and the results are reported as the means ± SD.

For comparison between the two groups, a *t*-test was used, and differences were considered to be significantly different if *p* < 0.05 (GraphPad PRISM 8.2). One-way analysis of variance (ANOVA) was carried out in GraphPad PRISM 8.2 software to evaluate any statistical difference among more than two different samples. Differences were considered to be significantly different if *p* < 0.05 (Tukey’s post-hoc multiple-comparison test).

## 5. Conclusions

The effect of natural compounds on glucose transporters and their protective effects against oxidative stress have been previously demonstrated in several experimental models. For the first time, this study highlights the ability of POE to reduce glucose transport by lowering the GLUT2 levels, to promote intestinal barrier integrity by modulating ZO-1 levels, and to have a protective antioxidant effect against glucose-induced damage in Caco-2 cells. This is the first study to investigate the behavior of a complex pool of POE phenolic compounds, rather than a single molecule, on differentiated human intestinal Caco-2 cells. We suggest that the biological properties exerted by POE could be related to synergistic effects of its constituents.

In the face of the incessant demand for new alternative natural antioxidants, as well as anti-inflammatory and antidiabetic agents, the cell-safe POE profile makes this phytocomplex an excellent candidate for continuing to investigate the potential use of POE in the management of chronic diseases, including diabetes, in support of practices described in traditional medicine. Therefore, our results lay the foundation for further in vitro and in vivo studies to verify the antioxidant and antidiabetic role of *P. oceanica*.

## Figures and Tables

**Table 1:** Biochemical properties of POE in terms of total polyphenols (TP) antioxidant (FRAP assay) and radical scavenging activities (DPPH assay).

**TP**	3.4 ± 0.2 mg GAE/mL
**FRAP**	0.9 ± 0.2 mg AAE/mL
**DPPH**	8.9 ± 0.3 mg AAE/mL

## Data Availability

Not applicable.

## Supplementary Materials

The following supporting information can be downloaded at: https://www.mdpi.com/article/10.3390/md20070457/s1, Figure S1: Effect of POE on Caco-2 cells viability; Figure S2: Effect of POE on HG-induced intracellular ROS formation; Figure S3: Effect of POE on HG-induced cell toxicity.
