# Supplementary material for: Glucose Uptake and Oxidative Stress in Caco-2 Cells: Health Benefits from Posidonia oceanica (L.) Delile
Source: Mar Drugs. 2022 Jul 14;20(7):457. doi: 10.3390/md20070457 (PMC9319946; doi:10.3390/md20070457)

marine drugs

Supplementary Material

Figure S1. Effect of POE on Caco-2 cells viability. Caco-2 cells were incubated in the absence (Control cells) or in the presence of increasing concentrations of POE (5-40 µg GAE/mL). Results are represented as mean ± SD of 5 determinations carried out in triplicate.

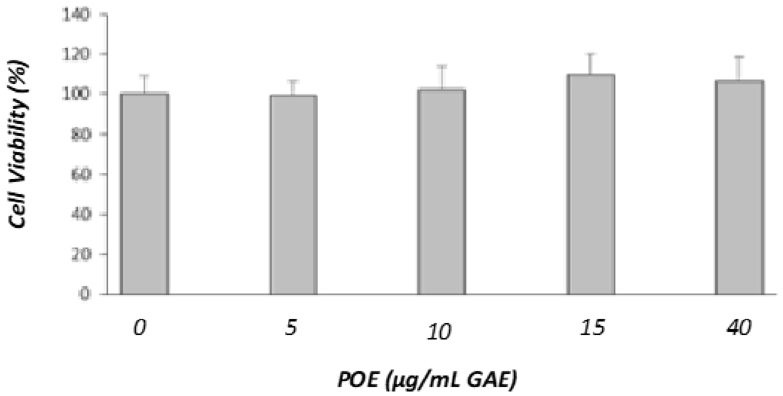

Figure S2. Effect of POE on HG-induced intracellular ROS formation. Intracellular ROS production was evaluated in Caco-2 cells incubated in the absence (Control cells) or in the presence of high-glucose condition (HG-cells) for 1 week and co-incubated with POE (0-40 µg GAE/mL), for the last 24h.Values are represented as mean ± SD of 4 determinations carried out in triplicate. ***Ctrl vs HG, *** HG vs HG+POE. (ANOVA, ***: p<0.001)

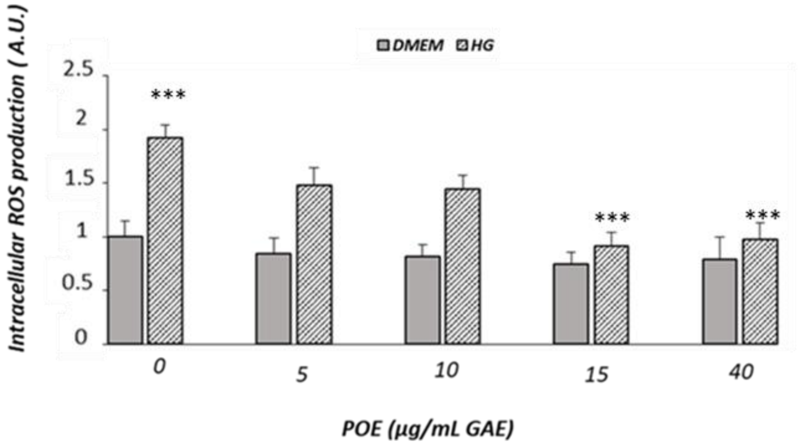

Figure S3. Effect of POE on HG-induced cell toxicity. Caco-2 cells incubated in the absence (Control cells) or in the presence of high-glucose condition (HG-cells) for 1 week and co-incubated with POE (15 µg GAE/mL). Results are represented as mean ± SD of 5 determinations carried out in triplicate. *: vs Control ($p < 0.05$)

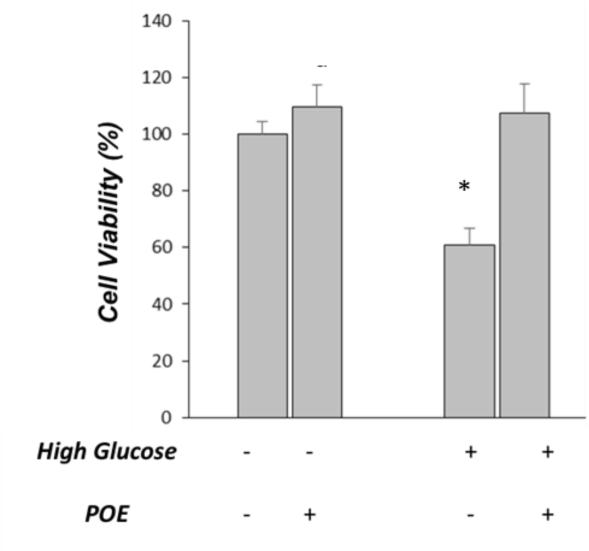

Supplement: Supplementary file 1 [file marinedrugs-20-00457-s001.zip › marinedrugs-1802722-supplementary.pdf]
